# Retraction: Moringa leaf extract improves biochemical attributes, yield and grain quality of rice (*Oryza sativa* L.) under drought stress

**DOI:** 10.1371/journal.pone.0272182

**Published:** 2022-08-03

**Authors:** The PLOS ONE Editors

The *PLOS ONE* Editors retract this article [1] because it was identified as one of a series of submissions for which we have concerns about authorship, competing interests, and peer review. We regret that the issues were not addressed prior to the article’s publication.

MBH, MMM, SI, and ZH either did not respond directly or could not be reached. SafdarB responded but expressed neither agreement nor disagreement with the editorial decision. AB, AMES, BSA, MSS, SaqibB, SK, and YL did not agree with the retraction.
